# Microbiota-Specific CD4CD8αα Tregs: Role in Intestinal Immune Homeostasis and Implications for IBD

**DOI:** 10.3389/fimmu.2015.00522

**Published:** 2015-10-08

**Authors:** Guillaume Sarrabayrouse, Joudy Alameddine, Frédéric Altare, Francine Jotereau

**Affiliations:** ^1^Digestive System Research Unit, University Hospital Vall d’Hebron, Barcelona, Spain; ^2^U892, INSERM, Nantes, France; ^3^Université de Nantes, Nantes, France; ^4^UMR 6299, CNRS, Nantes, France

**Keywords:** Tregs, *Faecalibacterium prausnitzii*, IBD, microbiota, inflammation

## Abstract

In studies in murine models, active suppression by IL-10-secreting Foxp3 regulatory T cells (Tregs) has emerged as an essential mechanism in colon homeostasis. However, the role of the equivalent subset in humans remains unclear, leading to suggestions that other subsets and/or mechanisms may substitute for Foxp3 Tregs in the maintenance of colon homeostasis. We recently described a new subset of CD4CD8αα T cells reactive to the gut bacterium *Faecalibacterium prausnitzii* and endowed with regulatory/suppressive functions. This subset is abundant in the healthy colonic mucosa, but less common in that of patients with inflammatory bowel disease (IBD). We discuss here the physiological significance and potential role of these Tregs in preventing inflammation of the gut mucosa and the potential applications of these discoveries for IBD management.

## Diversity of Peripherally Derived Tregs (pTregs)

CD4 regulatory T cells (Tregs) inhibit inflammatory responses (1). They can be subdivided into natural Tregs, which differentiate in the thymus (tTreg) and peripherally derived Tregs (pTregs), which differentiate in secondary lymphoid organs or tissues (2). These populations differ in terms of their non-redundant roles: tTregs play an essential role in maintaining tolerance toward self-structures, whereas pTregs are involved in the responses to externally delivered antigens or commensal microbes. Furthermore, the tTreg population appears to be stable, whereas that of pTregs may be more labile (3). This functional dichotomy results from differences in differentiation due to exposure to different TCR ligands (self and non-self antigens, respectively) and specific factors (cytokines, route of exposure, and antigen-presenting cells) in contrasting settings (4). The two Treg subsets can also be distinguished on the basis of the presence or absence of constitutive expression of the Foxp3 transcription factor. Constitutive Foxp3 expression and more particularly, the demethylation of a specific region of the Foxp3 locus are characteristic features of tTregs (5). Three main subsets of CD4 pTregs have been described in mice: Foxp3^+^CD25^+^ lymphocytes (3, 6), which are particularly abundant in the colon lamina propria (LP) (7) and two Foxp3^−^ subsets: the type 1 regulatory T (Tr1) cells and the T helper 3 (Th3) cells. The Tr1 subset secretes IL-10 and TGF-β in the absence of IL-4 and IL-17 (8–10) and is abundant in the small intestine (7). The Th3 subset may also secrete IL-10, but it differs from Tr1 in its expression of membrane-bound TGF-β (11, 12). The Tr1 Tregs are induced *in vitro* by IL-10 (8–10) and *in vivo* by TGF-β and IL-27 (9, 13) in the context of diverse immune responses (14) and upon chronic stimulation with antigens in the presence of IL-10 (10). The suppressive action of Tr1 Tregs is essentially IL-10-dependent, but it is also at least partly governed by TGF-β (8, 9). Moreover, the suppressive function of these cells may be mediated by a cytotoxic mechanism dependent on granzyme B and perforin (15). The Th3 subset is induced in the gut mucosa by oral immunization (12, 13). Its suppressive effects are essentially mediated by TGF-β, but also partly by IL-10 (11, 16). Much remains unknown about the typical features of Tr1 and Th3 cells and their relative contributions to immune regulation in general and to gut homeostasis in particular. Recent studies have shown that the development of colonic Foxp3^+^ Tregs in mice is induced by gut clostridial bacteria and their metabolites, and that these Tregs play a key role in the prevention of colitis (17, 18). In humans, however, the role of gut Foxp3^+^ Tregs in irritable bowel disease (IBD) remains unclear (19, 20), leading to suggestions that these cells may be less crucial in humans than in mice for the maintenance of colon homeostasis (21, 22).

## Human Colon DP8α T Cells are pTregs Induced by Clostridial Bacteria

We recently reported that the CD4CD8αα (DP8α) lymphocytes of the colon LP are Foxp3^−^ IL-10-secreting Tregs highly skewed toward the recognition of *Faecalibacterium prausnitzii*, a gut bacterium belonging to cluster IV of the genus *Clostridum* (23). In the healthy colonic mucosa of colon cancer patients, these cells account for about 12% of the CD4 lymphocytes present. We have shown that about 2% of the CD4 PBLs have the same CD4CD8αα phenotype and that 15% of these cells, on average, also react with *F. prausnitzii* (23). Together with the role of clostridial antigens in the induction of mouse colonic Tregs (17, 24) and the demonstration that segmented filamentous bacteria (SFB) antigens induce Th17 lymphocytes in the small intestine (25), our data suggest that *F. prausnitzii* participates in the induction of human DP8α colonic Tregs through antigen presentation. Support for this hypothesis is provided by our recent observation that *F. prausnitzii* imprints a phenotypic tolerogenic profile including a failure to secrete IL-12 on LPS-matured human DCs *in vitro* (unpublished data). Interestingly, *F. prausnitzii* is the most abundant bacterium of the human intestinal microbiota in healthy adults (26, 27) and decreases in its abundance have been linked to dysbiosis in IBD (28–32). The unique anti-inflammatory potential of this bacterium has recently been demonstrated, both *in vitro* and *in vivo* (33, 34). We found that there were fewer DP8α Tregs in the inflamed colonic mucosa and blood of Crohn’s disease patients and in the blood of ulcerative colitis (UC) patients than in healthy individuals (23). These results suggest that lower levels of *F. prausnitzii* are associated with lower levels of *F. prausnitzii*-specific Treg anti-inflammatory activity in IBD patients, and that this may contribute to the disease. As a corollary, this suggests that DP8α Tregs may play a role in colon homeostasis and IBD prevention. However, this hypothesis requires confirmation and a number of important questions about these cells remain to be answered, to define more precisely their contribution to IBD prevention.

## DP8α Treg: A New pTreg Subtype

We must first consider whether DP8α lymphocytes represent a new pTreg subtype. If Tr1 cells are defined as Foxp3^−^ Tregs secreting IL-10, then DP8α T cells could be considered to be Tr1 cells. Gagliani et al. (35) have suggested that human and mouse Tr1 cells are defined by the coexpression of CD49b and LAG3. We have also reported the expression of LAG3 by colonic DP8α cells *ex vivo* (23), but we did not consider their expression of CD49b. Nevertheless, our data revealed significant differences between Tr1 cells and DP8α Tregs. For example, DP8α Tregs stably express the CD8αα homodimer, CD25 and the transcription factor GATA-3, but do not express PD1, considered to be a canonical marker of Tr1 cells (9, 35). Moreover, whereas suppression by Tr1 and Th3 Tregs is largely dependent on IL-10 or TGF-β secretion, respectively (7, 9), the inhibition of T-cell proliferation by DP8α Tregs *in vitro* was little affected by a blocking anti-IL-10 antibody and not at all affected by an anti-TGF-β receptor antibody (23). It is, therefore, possible to distinguish DP8α Tregs from the Tr1 and Th3 Treg subsets.

One surprising finding of our work is the lack of Foxp3 expression by DP8α Tregs. However, they otherwise strongly resemble mouse Foxp3 colonic Tregs in terms of their regulatory markers (CD25, CTLA-4, GITR, and LAG3), regulatory functions (inhibition of T-cell proliferation, inhibition of DC maturation, and IL-10 secretion) and induction by related clostridial species (23). In both mice and humans, Foxp3 expression is required to maintain the Treg cell program and suppressive functions of tTreg (36) by repressing the activation-dependent expression of a number of genes, as elegantly shown in a recent study (37). In mice, Foxp3 is also expressed by the pTregs induced by clostridial bacteria (17). We have reported that DP8α Tregs have highly stable regulatory properties (23). This implies a high degree of commitment of these cells to their Treg status, with the expression of a Foxp3-independent genetic program in these cells. We are currently trying to decipher the genetic basis of DP8αα Treg commitment by comparing the transcriptomic signatures of the three main subtypes of CD4 lymphocytes in the colon LP: DP8α Tregs, conventional CD4 (CD4^+^CD25^−^CD127^High^), and Foxp3 Tregs (CD4^+^CD25^+^CD127^low^), with and without activation.

## Colonic DP8α Tregs: Functional Homologs of the pTregs Induced by Clostridial Bacteria in Mice?

**Figure 1:**
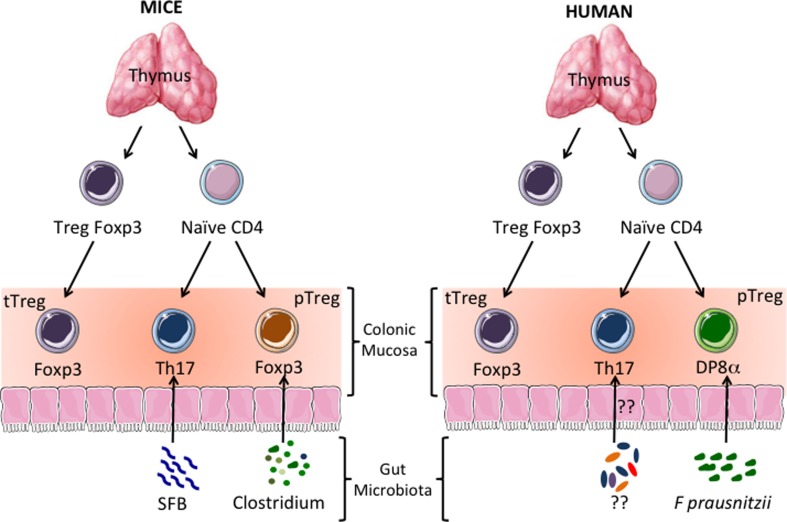
**Human CD4CD8αα (DP8α) regulatory T cells (Treg): functional homologs of the Tregs induced by clostridial bacteria in mice?** In mice and humans, a subpopulation of CD4^+^ thymocytes develops into Tregs expressing the transcription factor Foxp3. These thymus-derived Tregs (tTregs) migrate to all tissues, including the colonic mucosa, where they prevent autoimmune reactions. Thymus-derived naïve CD4^+^ T cells also migrate to the colonic mucosa. There, depending on the type of antigen-dependent signals they receive, they develop into effector lymphocytes or peripherally induced Tregs (pTregs). It has been shown in mice that colonic Th17 and Foxp3^+^ IL-10-secreting pTregs are induced by segmented filamentous bacteria (SFB) and clostridial bacteria, respectively. In the human colonic mucosa, IL-10-secreting DP8α Tregs induced by *Faecalibacterium prausnitzii* (*F. prausnitzii*), a gut bacterium belonging to the *Clostridium* cluster IV, seem to be the homologs of the mouse Foxp3 pTregs.

One important question raised by our results is whether human DP8α Tregs are functional homologs of the mouse Foxp3 Tregs induced by clostridial species (17), as shown in Figure 1. Alternatively, clostridial bacteria might induce both Treg subsets, with these subset playing complementary roles in colon homeostasis. This second hypothesis is unlikely in mice, because most of the IL-10-secreting Tregs of the colon LP express Foxp3, so IL-10-secreting Foxp3-negative lymphocytes are missing from this compartment (7). CD4CD8αα IL-10-secreting T lymphocytes have been described in the mouse gut mucosa. However, these cells were located in the epithelium of the small intestine (38), a compartment clearly different from the colon in terms of the composition and function of its immune components (39). In addition, we found no reactivity to *F. prausnitzii* in freshly sorted human colonic Foxp3 Tregs, suggesting that this bacterium (or at least its antigens) is not involved in the induction of Foxp3 lymphocytes in the human colon LP (unpublished data). This may appear to conflict with the induction of Foxp3 Treg development by human clostridial bacteria in the colonic mucosa of GF mice (24), but the true meaning of this result remains unclear, because another study has shown that the human microbiota cannot restore normal mouse colon development upon transfer into GF animals (40). It, therefore, appears possible that during evolution, both humans and rodents have selected clostridial symbionts on the basis of their capacity to maintain colon homeostasis via Treg induction, but that these two groups diverged in terms of the molecular mechanisms involved in this process. Consistent with the hypothesis that DP8α Tregs may be functional homologs of mouse Foxp3 pTregs, the role of human Foxp3 Tregs in the prevention of colitis remain unclear (19). Moreover, the manifestations of enteropathy in IPEX (immune dysregulation, polyendocrinopathy, enteropathy, X-linked syndrome) patients (who lack functional Foxp3 Treg), which are often considered to provide support for a role for Foxp3^+^ Tregs in the prevention of IBD, clearly differ from those in IBD (41). This suggests that IPEX-associated colitis results from autoimmune attacks rather than from defects in tolerance to the microbiota and, thus, that mechanisms other than Foxp3 Treg-dependent suppression, possibly including suppression by DP8α Tregs, are involved in human colonic homeostasis (21, 22).

## Are all Blood DP8α Lymphocytes Regulatory T Cells?

About 2% of CD4 PBLs have the same double-positive phenotype as DP8α LPLs, raising questions about their function. Most DP8α PBLs lacked regulatory markers *ex vivo*, but they acquired these markers and regulatory functions after a short period of *in vitro* activation or establishment in culture (which also requires TCR activation), whereas their CD4 homologs did not. Moreover, *ex vivo*, about 10% of DP8α PBLs expressed the gut homing receptor CCR9, and about the same proportion recognized *F. prausnitzii* (23). Therefore, most DP8α PBLs appear to be Tregs, although only a limited fraction of these cells react to *F. prausnitzii*. It is possible that some of these cells are pTregs induced by microbiota components present outside the gut, in the pulmonary mucosa, or the skin, for example. It is also possible that some of the circulating DP8α lymphocytes are not Tregs. Additional studies will be required to address these questions and to determine the specificity of the TCRs of regulatory DP8α PBLs that do not recognize *F. prausnitzii*.

## Does the CD8αα Molecule Play a Role in DP8α Treg Function?

CD8αα expression can be transiently induced on human CD4^+^ T lymphocytes by activation in the presence of IL-4 (42). However, this molecule is expressed constitutively by the DP8α lymphocytes of the human colon LP and blood (23). This raises questions about the possible role of this molecule in DP8α Treg function. Like the CD8αβ coreceptor, CD8αα binds to MHC class-I molecules (43). In mice and humans, it also binds to the specific ligands thymus leukemia antigen (TL) (44) and gp180 (CEACAM5)/CD1d (45, 46), respectively. The human CD8αα ligand is expressed in the gut, by non-lymphoid cells, such as colonic epithelial cells and polynuclear neutrophils, two types of non-professional APCs that can activate MHC class II-restricted T lymphocytes. Previous studies have shown that the interaction between the CD8αα molecule and its ligands costimulates both the TCR activation induced by specific MHC/Ag complexes or by CD3 antibody, and T-cell function (44, 47). Moreover, CD8αα has been shown to play a role in the selection of high-affinity CD8αβ T cells (48). We have observed (unpublished data) that the triggering of CD8αα by an anti-CD8 antibody (OKT8) enhances the activation of DP8α Tregs induced by an anti-CD3 antibody (OKT3). This suggests that ligation of the CD8αα molecule of DP8α Tregs may increase their TCR-dependent activation by microbiota-derived antigens. It has recently been shown that TCR signaling is critical for the maintenance of the suppressive capacity of Foxp3 Tregs in mice, particularly in the colonic mucosa (49). It would be interesting to determine whether there is a similar dependence on TCR signaling in DP8α Tregs and whether the CD8αα receptor contributes to this process.

## DP8α Tregs, Biological Markers and Therapeutic Targets in IBD

**Figure 2:**
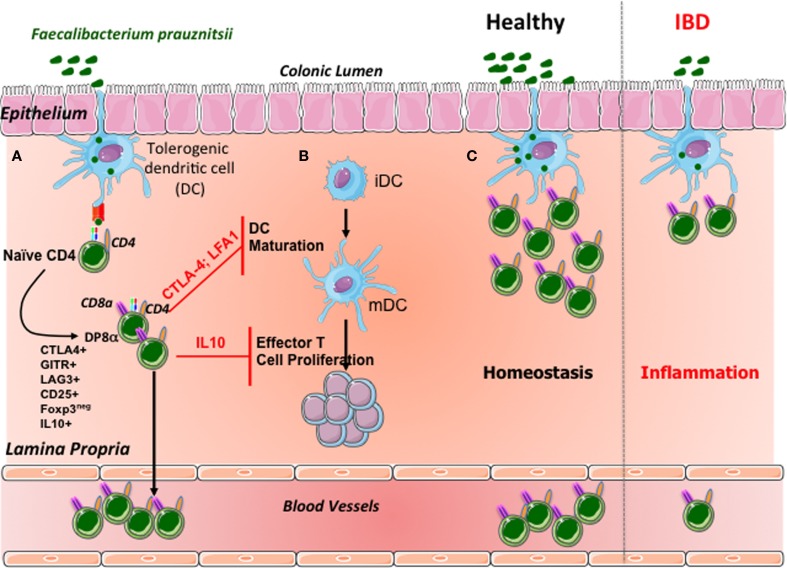
**Suggested model of DP8α Treg induction and homeostasis control in the human colonic mucosa**. **(A)** In the colonic mucosa, *F. prausnitzii* antigens are presented by dendritic cells. Simultaneously, these bacteria may imprint a tolerogenic phenotype on dendritic cells. The recognition of *F. prausnitzii* antigens by the naïve CD4 T cells equipped with a specific TCR contributes, in this context, to the differentiation of these cells into Foxp3-lacking Tregs coexpressing CD4 and CD8α. DP8α T lymphocytes express most of the regulatory markers of Foxp3 Tregs and secrete IL-10. A fraction of these cells migrate into the blood. **(B)** Like Foxp3 Tregs, DP8α Tregs inhibit the maturation of dendritic cells in a CTLA-4- and LFA-1-dependent manner. Moreover, these cells decrease effector T-cell proliferation, via a mechanism involving interleukin 10. **(C)** Under normal circumstances, colonic *F. prausnitzii* expands the pool of DP8α Tregs in the colonic mucosa and in the blood; in IBD patients, the low levels of *F. prausnitzii* may compromise the expansion or survival of the DP8α Treg population, reducing the frequency of these cells in the mucosa and blood of patients.

Our observation that DP8α Treg levels are low in the colonic LP and blood of IBD patients, who frequently also have low levels of *F. prausnitzii* in their gut microbiota, provides evidence in support of a correlation between the levels of these bacteria and DP8α Treg levels in these patients (Figure 2). As recently suggested (50), such a correlation might result from a feedback loop between the selection, by follicular regulatory T cells (Tfr), of an adequate IgA repertoire fostering microbiota diversity, particularly as concerns the abundance of clostridial bacteria, which in turn govern the development or survival of DP8α Tregs. In this context, the possible presence of DP8α Tfr should be investigated.

It is currently difficult to determine whether there is a strong correlation between the levels of DP8α Tregs and *F. prausnitzii* in the colonic mucosa, as no method for quantifying DP8α lymphocytes in biopsy specimens is available. There is an urgent need to develop such a method, based on CD4 and CD8α colabeling by immunohistochemistry, although this approach would not distinguish between CD4CD8αα and CD4CD8αβ lymphocytes, or, preferentially, quantitative RT-PCR, if a specific marker of colonic DP8α lymphocytes can be identified from the transcriptomic signature of these cells. Efforts are currently being made to identify such a marker.

Only about 15% of DP8α PBLs appear to be specific for *F. prausnitzii*, suggesting that the remaining circulating DP8α lymphocytes are not induced by the gut microbiota. Nevertheless, the total frequency of DP8α PBL and the frequency of these cells for *F. prausnitzii* are lower in the blood of IBD patients than in controls (23). The question as to whether the frequency of circulating DP8α lymphocytes and/or of DP8α lymphocytes reactive to *F. prausnitzii* can be viewed as a biological marker of IBD is an important issue as there are currently no specific biomarkers of this disease. It will be necessary to determine whether DP8α levels are correlated with disease type and activity and predict disease progression in a large cohort of IBD patients to answer this question.

If DP8α levels in the blood or the colonic mucosa are found to be predictive of disease progression, this would provide an objective means of assessing the contribution of these Tregs to the prevention of IBD. Such an advance would open up new possibilities for treating IBD by manipulating the frequency of *F. prausnitzii* in the gut microbiota or increasing the number of circulating DP8α Tregs through specific *in vivo* stimulation or induction, or adoptive transfers of these cells. We have found that DP8α Tregs proliferate well *in vitro*, whilst maintaining their regulatory phenotype and functions (23).

## Concluding Remarks – Future Orientations

We have identified, for the first time in humans, a mechanism by which the gut microbiota can affect gut homeostasis: the induction of DP8α Tregs in a mucosa exposed to frequent stimulation with microbiota-derived immune stimuli, both PAMPs and microbe antigens. The precise physiological significance of DP8α Tregs remains to be determined, but the discovery of these cells has potentially wide-ranging implications for the management of IBD and, potentially, of other immune diseases involving the abnormal induction and/or function of microbiota-induced DP8α Tregs.

## Conflict of Interest Statement

The authors declare that the research was conducted in the absence of any commercial or financial relationships that could be construed as a potential conflict of interest.

## References

[1] Josefowicz SZ Lu LF Rudensky AY Regulatory T cells: mechanisms of differentiation and function. Annu Rev Immunol (2007) 30:531–64. 10.1146/annurev.immunol.25.022106.141623 22224781 PMC6066374

[2] Abbas AK Benoist C Bluestone JA Campbell DJ Ghosh S Hori S Regulatory T cells: recommendations to simplify the nomenclature. Nat Immunol (2013) 14:307–8. 10.1038/ni.2554 23507634

[3] Shevach EM Thornton AM. tTregs, pTregs, and iTregs: similarities and differences. Immunol Rev (2014) 259:88–102. 10.1111/imr.12160 24712461 PMC3982187

[4] Weissler KA Caton AJ. The role of T-cell receptor recognition of peptide:MHC complexes in the formation and activity of Foxp3(+) regulatory T cells. Immunol Rev (2014) 259:11–22. 10.1111/imr.12177 24712456 PMC4034456

[5] Wieczorek G Asemissen A Model F Turbachova I Gloess S Liebenberg V Quantitative DNA methylation analysis of FOXP3 as a new method for counting regulatory cells in peripheral blood and solid tissue. Cancer Res (2009) 15:599–608. 10.1158/0008-5472.CAN-08-2361 19147574

[6] Yadav M Stephan S Bluestone JA. Peripherally induced tregs – role in immune homeostasis and autoimmunity. Front Immunol (2013) 4:232. 10.3389/fimmu.2013.00232 23966994 PMC3736167

[7] Maynard CL Harrington LE Janowski KM Oliver JR Zindl CL Rudensky AY Regulatory T cells expressing interleukin 10 develop from Foxp3^+^ and Foxp3^-^ precursor cells in the absence of interleukin 10. Nat Immunol (2007) 8:931–41. 10.1038/ni1504 17694059

[8] Groux H O’Garra A Bigler M Rouleau M Antonenko S de Vries JE A CD4^+^ T-cell subset inhibits antigen-specific T-cell responses and prevents colitis. Nature (1997) 389:737–42. 10.1038/39614 9338786

[9] Gregori S Goudy KS Roncarolo MG. The cellular and molecular mechanisms of immuno-suppression by human type 1 regulatory T cells. Front Immunol (2012) 3:30. 10.3389/fimmu.2012.00030 22566914 PMC3342353

[10] Roncarolo MG Gregori S Bacchetta R Battaglia M. Tr1 cells and the counter-regulation of immunity: natural mechanisms and therapeutic applications. Curr Top Microbiol Immunol (2014) 380:39–68. 10.1007/978-3-662-43492-5_3 25004813

[11] Chen Y Kuchroo VK Inobe J Hafler DA Weiner HL Regulatory T cell clones induced by oral tolerance: suppression of autoimmune encephalomyelitis. Science (1994) 265:1237–40. 10.1126/science.7520605 7520605

[12] Ochi H Abraham M Ishikawa H Frenkel D Yang K Basso AS Oral CD3-specific antibody suppresses autoimmune encephalomyelitis by inducing CD4^+^ CD25^-^ LAP^+^ T cells. Nat Med (2006) 12:627–35. 10.1038/nm1408 16715091

[13] Awasthi A Carrier Y Peron JP Bettelli E Kamanaka M Flavell RA A dominant function for interleukin 27 in generating interleukin 10-producing anti-inflammatory T cells. Nat Immunol (2007) 8:1380–9. 10.1038/ni1541 17994022

[14] Hawrylowicz CM O’Garra A. Potential role of interleukin-10-secreting regulatory T cells in allergy and asthma. Nat Rev Immunol (2005) 5:271–83. 10.1038/nri1589 15775993

[15] Magnani CF Alberigo G Bacchetta R Serafini G Andreani M Roncarolo MG Killing of myeloid APCs via HLA class I, CD2 and CD226 defines a novel mechanism of suppression by human Tr1 cells. Eur J Immunol (2011) 6:1652–62. 10.1002/eji.201041120 21469116 PMC3116154

[16] Gandhi R Farez MF Wang Y Kozoriz D Quintana FJ Weiner HL. Cutting edge: human latency-associated peptide+ T cells: a novel regulatory T cell subset. J Immunol (2010) 184:4620–4. 10.4049/jimmunol.0903329 20368276 PMC2904991

[17] Atarashi K Tanoue T Shima T Imaoka A Kuwahara T Momose Y Induction of colonic regulatory T cells by indigenous *Clostridium* species. Science (2011) 331:337–41. 10.1126/science.1198469 21205640 PMC3969237

[18] Kugelberg E Mucosal immunology: bacteria get T(Reg) cells into shape. Nat Rev Immunol (2014) 14:2–3. 10.1038/nri3794 24287865

[19] Maul J Loddenkemper C Mundt P Berg E Giese T Stallmach A Peripheral and intestinal regulatory CD4*+* CD25(high) T cells in inflammatory bowel disease. Gastroenterology (2005) 128:1868–78. 10.1053/j.gastro.2005.03.043 15940622

[20] Buckner JH. Mechanisms of impaired regulation by CD4(+)CD25(+)FOXP3(+) regulatory T cells in human autoimmune diseases. Nat Rev Immunol (2010) 10:849–59. 10.1038/nri2889 21107346 PMC3046807

[21] Tanoue T Honda K. Induction of Treg cells in the mouse colonic mucosa: a central mechanism to maintain host-microbiota homeostasis. Semin Immunol (2012) 24:50–7. 10.1016/j.smim.2011.11.009 22172550

[22] MacDonald TT Monteleone I Fantini MC Monteleone G. Regulation of homeostasis and inflammation in the intestine. Gastroenterology (2011) 140:1768–75. 10.1053/j.gastro.2011.02.047 21530743

[23] Sarrabayrouse G Bossard C Chauvin JM Jarry A Meurette G Quevrain E CD4CD8alphaalpha lymphocytes, a novel human regulatory T cell subset induced by colonic bacteria and deficient in patients with inflammatory bowel disease. PLoS Biol (2014) 12:e1001833. 10.1371/journal.pbio.1001833 24714093 PMC3979654

[24] Atarashi K Tanoue T Oshima K Suda W Nagano Y Nishikawa H Treg induction by a rationally selected mixture of *Clostridia* strains from the human microbiota. Nature (2013) 500:232–6. 10.1038/nature12331 23842501

[25] Goto Y Panea C Nakato G Cebula A Lee C Diez MG Segmented filamentous bacteria antigens presented by intestinal dendritic cells drive mucosal Th17 cell differentiation. Immunity (2014) 40:594–607. 10.1016/j.immuni.2014.03.005 24684957 PMC4084624

[26] Hold GL Schwiertz A Aminov RI Blaut M Flint HL. Oligonucleotide probes that detect quantitatively significant groups of butyrate-producing bacteria in human feces. Appl Environ Microbiol (2003) 7:4320–4. 10.1128/AEM.69.7.4320-4324.2003 12839823 PMC165216

[27] Miquel S Martin R Rossi O Bermudez-Humaran LG Chatel JM Sokol H *Faecalibacterium prausnitzii* and human intestinal health. Curr Opin Microbiol (2013) 16:255–61. 10.1016/j.mib.2013.06.003 23831042

[28] Sokol H Pigneur B Watterlot L Lakhdari O Bermudez-Humaran LG Gratadoux JJ F*aecalibacterium prausnitzii* is an anti-inflammatory commensal bacterium identified by gut microbiota analysis of Crohn disease patients. Proc Natl Acad Sci U S A (2008) 105:16731–6. 10.1073/pnas.0804812105 18936492 PMC2575488

[29] Willing B Halfvarson J Dicksved J Rosenquist M Jarnerot G Engstrand L Twin studies reveal specific imbalances in the mucosa-associated microbiota of patients with ileal Crohn’s disease. Inflamm Bowel Dis (2009) 15:653–60. 10.1002/ibd.20783 19023901

[30] Manichanh C Borruel N Casellas F Guarner F. The gut microbiota in IBD. Nat Rev Gastroenterol Hepatol (2012) 9:599–608. 10.1038/nrgastro.2012.152 22907164

[31] Sokol H Seksik P Furet JP Firmesse O Nion-Larmurier I Beaugerie L Low counts of *Faecalibacterium prausnitzii* in colitis microbiota. Inflamm Bowel Dis (2009) 15:1183–9. 10.1002/ibd.20903 19235886

[32] Machiels K Joossens M Sabino J De Preter V Arijs I Eeckhaut V A decrease of the butyrate-producing species *Roseburia hominis* and *Faecalibacterium prausnitzii* defines dysbiosis in patients with ulcerative colitis. Gut (2013) 63:1275–83. 10.1136/gutjnl-2013-304833 24021287

[33] Martin R Chain F Miquel S Lu J Gratadoux JJ Sokol H The commensal bacterium *Faecalibacterium prausnitzii* is protective in DNBS-induced chronic moderate and severe colitis model. Inflamm Bowel Dis (2014) 20:417–30. 10.1097/01.MIB.0000440815.76627.64 24418903

[34] Quévrain E Maubert MA Michon C Chain F Marquant R Tailhades J Identification of an anti-inflammatory protein from *Faecalibacterium prausnitzii*, a commensal bacterium deficient in Crohn’s disease. Gut (2015). 10.1136/gutjnl-2014-307649 26045134 PMC5136800

[35] Gagliani N Magnani CF Huber S Gianolini ME Pala M Licona-Limon P Coexpression of CD49b and LAG-3 identifies human and mouse T regulatory type 1 cells. Nat Med (2013) 19:739–46. 10.1038/nm.3179 23624599

[36] Fontenot JD Gavin MA Rudensky AY. Foxp3 programs the development and function of CD4^+^CD25^+^ regulatory T cells. Nat Immunol (2003) 4:330–6. 10.1038/ni904 12612578

[37] Arvey A van der Veeken J Samstein RM Feng Y Stamatoyannopoulos JA Rudensky AY. Inflammation-induced repression of chromatin bound by the transcription factor Foxp3 in regulatory T cells. Nat Immunol (2007) 15:580–7. 10.1038/ni.2868 24728351 PMC4112080

[38] Das G Augustine MM Das J Bottomly K Ray P Ray A. An important regulatory role for CD4^+^CD8 alpha alpha T cells in the intestinal epithelial layer in the prevention of inflammatory bowel disease. Proc Natl Acad Sci U S A (2003) 100:5324–9. 10.1073/pnas.0831037100 12695566 PMC154344

[39] Mowat AM Agace WW Regional specialization within the intestinal immune system. Nat Rev Immunol (2014) 14:667–85. 10.1038/nri3738 25234148

[40] Chung H Pamp SJ Hill JA Surana NK Edelman SM Troy EB Gut immune maturation depends on colonization with a host-specific microbiota. Cell (2012) 149:1578–93. 10.1016/j.cell.2012.04.037 22726443 PMC3442780

[41] Patey-Mariaud de Serre N Canioni D Ganousse S Rieux-Laucat F Goulet O Ruemmele F Digestive histopathological presentation of IPEX syndrome. Mod Pathol (2009) 22:95–102. 10.1038/modpathol.2008.161 18820676

[42] Paliard X Malefijt RW de Vries JE Spits H. Interleukin-4 mediates CD8 induction on human CD4^+^ T-cell clones. Nature (1988) 335:642–4. 10.1038/335642a0 3262830

[43] Rybakin V Clamme JP Ampudia J Yachi PP Gascoigne NR. CD8alphaalpha and -alphabeta isotypes are equally recruited to the immunological synapse through their ability to bind to MHC class I. EMBO Rep (2011) 12:1251–6. 10.1038/embor.2011.209 22081144 PMC3245696

[44] Leishman AJ Naidenko OV Attinger A Koning F Lena CJ Xiong Y T cell responses modulated through interaction between CD8alphaalpha and the nonclassical MHC class I molecule, TL. Science (2001) 294:1936–9. 10.1126/science.1063564 11729321

[45] Campbell NA Park MS Toy LS Yio XY Devine L Kavathas P A non-class I MHC intestinal epithelial surface glycoprotein, gp180, binds to CD8. Clin Immunol (2002) 102:267–74. 10.1006/clim.2001.5170 11890713

[46] Roda G Jianyu X Park MS DeMarte L Hovhannisyan Z Couri R Characterizing CEACAM5 interaction with CD8alpha and CD1d in intestinal homeostasis. Mucosal Immunol (2014) 7:615–24. 10.1038/mi.2013.80 24104458 PMC3981948

[47] Allez M Brimnes J Shao L Dotan I Nakazawa A Mayer L. Activation of a unique population of CD8(+) T cells by intestinal epithelial cells. Ann N Y Acad Sci (2004) 1029:22–35. 10.1196/annals.1309.004 15681740

[48] Huang Y Park Y Wang-Zhu Y Larange A Arens R Bernardo I Mucosal memory CD8(+) T cells are selected in the periphery by an MHC class I molecule. Nat Immunol (2011) 12:1086–95. 10.1038/ni.2106 21964609 PMC3197978

[49] Levine AG Arvey A Jin W Rudensky AY. Continuous requirement for the TCR in regulatory T cell function. Nat Immunol (2010) 15:1070–8. 10.1038/ni.3004 25263123 PMC4205268

[50] Kawamoto S Maruya M Kato LM Suda W Atarashi K Doi Y Foxp3(+) T cells regulate immunoglobulinA selection and facilitate diversification of bacterial species responsible for immune homeostasis. Immunity (2014) 41:152–65. 10.1016/j.immuni.2014.05.016 25017466

